# Photocatalytic Crystalline and Amorphous TiO_2_ Nanotubes Prepared by Electrospinning and Atomic Layer Deposition

**DOI:** 10.3390/molecules26195917

**Published:** 2021-09-29

**Authors:** Orsolya Kéri, Eszter Kocsis, Dániel Attila Karajz, Zsombor Kristóf Nagy, Bence Parditka, Zoltán Erdélyi, Anna Szabó, Klára Hernádi, Imre Miklós Szilágyi

**Affiliations:** 1 Department of Inorganic and Analytical Chemistry, Budapest University of Technology and Economics, H-1111 Budapest, Hungary; orsolyakeri@gmail.com (O.K.); kocsis.eszti125@gmail.com (E.K.); karda412@gmail.com (D.A.K.); 2 Department of Organic Chemistry and Technology, Budapest University of Technology and Economics, H-1111 Budapest, Hungary; zsknagy@oct.bme.hu; 3 Department of Solid State Physics, Faculty of Sciences and Technology, University of Debrecen, H-4026 Debrecen, Hungary; parditka.bence@science.unideb.hu (B.P.); zoltan.erdelyi@science.unideb.hu (Z.E.); 4 Department of Applied and Environmental Chemistry, University of Szeged, H-6720 Szeged, Hungary; anna.szabo992@gmail.com (A.S.); hernadi@chem.u-szeged.hu (K.H.); 5 Institute of Physical Metallurgy, Metalforming and Nanotechnology, Faculty of Materials Science and Engineering, University of Miskolc, H-3515 Miskolc, Hungary

**Keywords:** electrospinning, ALD, TiO_2_, amorphous, nanotube, photocatalysis

## Abstract

In this work core/shell composite polymer/TiO_2_ nanofibers and from those TiO_2_ nanotubes were prepared. First, poly(vinyl alcohol) (PVA) and poly(vinylpyrrolidone) (PVP) fibers were synthetized by electrospinning. They were covered with a 100 nm thick amorphous TiO_2_ layer by atomic layer deposition at 50 °C. Later the polymer core was removed by two different methods: dissolution and annealing. In the case of dissolution in water, the as-prepared TiO_2_ nanotubes remained amorphous, while when annealing was used to remove the polymers, the TiO_2_ crystallized in anatase form. Due to this, the properties of amorphous and crystalline TiO_2_ nanotubes with exactly the same structure and morphology could be compared. The samples were investigated by SEM-EDX, ATR-IR, UV-Vis, XRD and TG/DTA-MS. Finally, the photocatalytic properties of the TiO_2_ nanotubes were studied by decomposing methyl-orange dye under UV light. According to the results, crystalline anatase TiO_2_ nanotubes reached the photocatalytic performance of P25, while amorphous TiO_2_ nanotubes had observable photocatalytic activity.

## 1. Introduction

Nowadays the importance of utilizing renewable energy sources is increasing. One of these renewable sources is sunlight, which can be converted into chemical energy for example through photocatalysis [1]. Photocatalysis uses solar energy to enable or accelerate chemical reactions [2]. The research of semiconductor oxide (e.g., WO_3_, ZnO or TiO_2_) photocatalysis has grown exponentially in the recent years [3]. The most widely used semiconductor oxide in this field is TiO_2_, which has three crystalline phases (anatase, rutile and brookite). The anatase and rutile forms have been thoroughly studied, and it was found that generally anatase TiO_2_ has a higher activity than rutile, mostly thanks to its higher adsorbance capacity for organic compounds [4,5]. Meanwhile brookite was not investigated as exhaustively, since it is immensely difficult to obtain in pure phase and due to this, it would be too expensive to use [6]. In amorphous form TiO_2_ is considered not to have any photocatalytic effect, owing to that its crystal structure contains many imperfections, which can serve as recombination centers for the electron-hole pairs. Amorphous TiO_2_ was only used in composition with nanostructured materials (e.g., lotus leaves [7], fullerene [8]) or with other photocatalitically active semiconductor oxides [9] to prepare photocatalysts. TiO_2_ and composites containing TiO_2_ are tested as photocatalysts for many different applications, amongst these are the decomposition of organic contaminants [10,11], water purification [12,13], the preparation of self-cleaning surfaces [14,15], sterilization [16,17], photoelectrochemical conversion [18,19], etc.

The photocatalytic efficiency is dependent on many properties of the material, such as the composition, crystallinity, morphology and the specific surface area. Thus, being able to control these characteristics is key in achieving good photocatalytic activity. These could be controlled through choosing the most suitable synthesis method. There are many different preparation strategies and techniques, which can be used or also combined to attain the best results [20,21]. One synthesis method, which enables the preparation of nanomaterials with high specific surface area and precisely controlled composition, is electrospinning [22,23]. This technique has a simple set-up; a polymer solution or melt, which can also contain the precursor salt of metal-oxides, is filled into a plastic syringe with a metal needle tip. Then high voltage is applied to it, which causes the eruption of charged jets from the surface of the liquid. These jets stretch to ultrathin fibers due to the applied electric field and dry mid-flight, before reaching the grounded collector. If the polymer solution already contains the precursor of a semiconductor oxide, e.g., titanium tetraisopropoxide, pure oxide nanofibers can be prepared by annealing the composite to high temperatures [24]. In this case the polymer content is burnt out completely.

If pure polymer fibers are prepared, they can be used as templates for the preparation of core/shell nanofibers by covering them with a thin layer of semiconductor oxide [25,26]. The polymers are usually thermosensitive materials, and due to that the deposition of the film onto them has to be done at low temperatures, but also a highly organized structure is needed for good photocatalytic activity. A thin film deposition method, that meets both requirements, is atomic layer deposition (ALD) [27,28]. By this method the growth of thin layers is possible within nanometer range precision, owing to the alternating surface controlled reactions. If the right precursors are chosen for the synthesis, the reaction temperature can be lowered even to room temperature; thus, it can be easily combined with electrospinning [29].

The aim of our work was to prepare polymer/TiO_2_ core/shell composite nanofibers and then TiO_2_ nanotubes by removing the polymer core. For that, first, poly(vinyl alcohol) (PVA) and poly(vinylpyrrolidone) (PVP) nanofibers were prepared by electrospinning from their aqueous solutions. These fibers were then used as templates for preparing polymer/TiO_2_ core/shell nanocomposites. The polymers were covered with amorphous TiO_2_ layers by ALD. For photocatalytic purposes crystalline TiO_2_ was needed, therefore the composite nanofibers were annealed to remove the polymer core and crystallize the TiO_2_ in anatase form. The polymer was also removed from the samples by dissolution, to be able to compare the properties of the anatase TiO_2_ nanotubes to amorphous ones that have the exact same morphology. All the samples were investigated by scanning electron microscopy (SEM), energy dispersive X-ray analysis (EDX), ATR-IR and UV-Vis spectroscopy and X-ray powder diffraction (XRD). Finally, the photocatalytic properties were studied by decomposing methyl orange dye under UV irradiation, which was followed by UV-Vis spectroscopy.

## 2. Results

### 2.1. Preparation of the Polymer Fibers

#### 2.1.1. SEM-EDX of the Polymer Nanofibers

**Figure 1:**
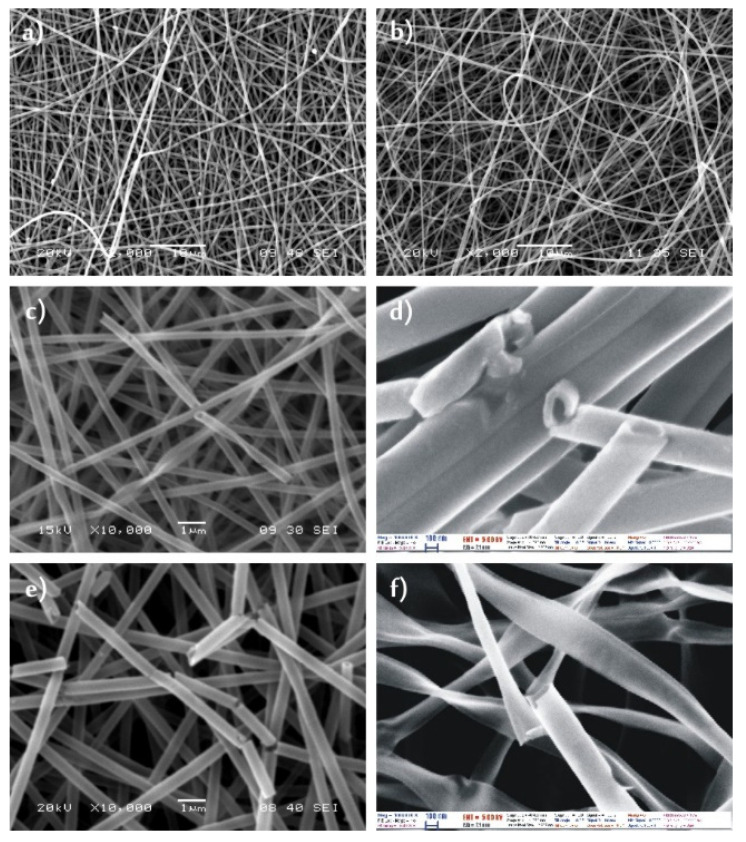
SEM images of (**a**) PVA nanofibers; (**b**) PVA/TiO_2_ composite nanofibers, (**c**,**d**) TiO_2_ nanotubes obtained from the PVA/TiO_2_ composite by dissolution, (**e**,**f**) TiO_2_ nanotubes obtained from the PVA/TiO_2_ composite by annealing.

**Figure 2:**
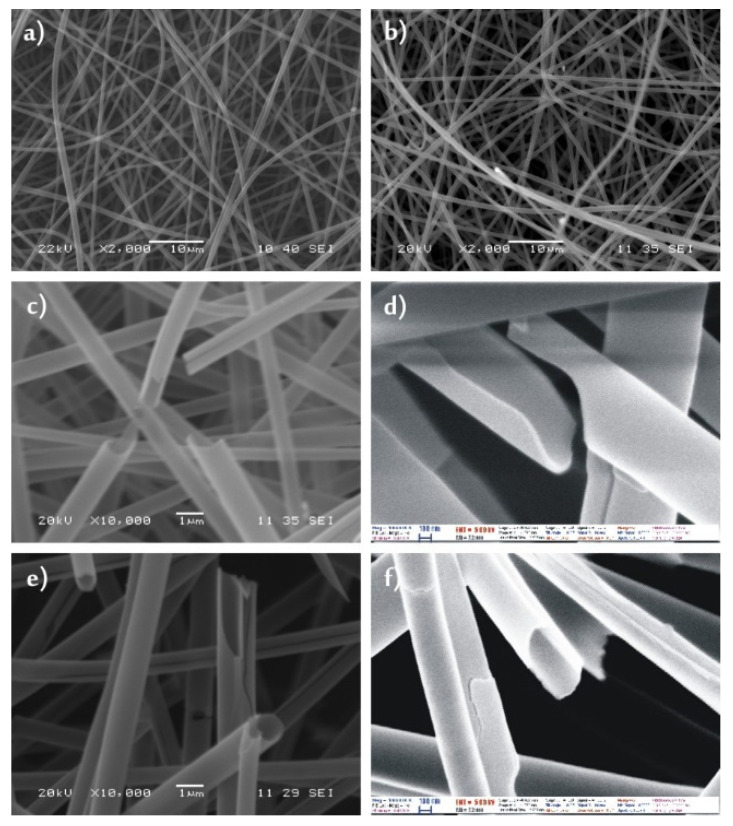
SEM images of (**a**) PVP nanofibers; (**b**) PVP/TiO_2_ composite nanofibers, (**c**,**d**) TiO_2_ nanotubes obtained from the PVP/TiO_2_ composite by dissolution, (**e**,**f**) TiO_2_ nanotubes obtained from the PVP/TiO_2_ composite by annealing.

The SEM images showed that both the as-prepared PVA (Figure 1a) and the PVP (Figure 2a) nanofibers had a fairly uniform fibrous structure, the fibers were several centimeters long. The PVA nanofibers had a diameter of 200–300 nm, while the PVP fibers were about 500–700 nm thick.

#### 2.1.2. Thermal Analysis of the Polymer Nanofibers in Nitrogen

**Figure 3:**
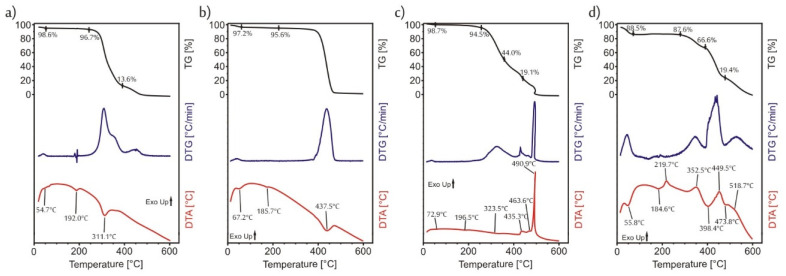
TG/DTA measurements, (**a**) PVA in N_2_; (**b**) PVP in N_2_; (**c**) PVA in air; (**d**) PVP in air.

Before any further synthesis steps, the thermal analysis of the fibers was done by TG/DTA (Figure 3) in inert (nitrogen) atmosphere in order to determine what temperature could be used for the ALD deposition without degrading the polymer fibers. In the case of both polymers, until 100 °C, there was no significant mass loss (Figure 3a,b); only water, which was used as the solvent during the electrospinning, evaporated. Then, above 200 °C the degradation of the polymers stared. The PVP decomposed in one step, while for the PVA the degradation consisted of several smaller overlapping steps. On the TGA curves of the PVA and the PVP as well, there were three endothermic peaks. The first one referred to the evaporation of the solvent, while the last one to the decomposition. When the second endothermic peak appeared on the DTA curve, there was no corresponding mass loss on the TG curves, so these endothermic peaks represented the softening of the PVA (at 192 °C) and of the PVP (at 186 °C). Based on these results, a very low temperature, 50 °C was chosen for the ALD depositions.

### 2.2. Preparation of the TiO_2_ Nanotubes

#### 2.2.1. SEM-EDX of the Polymer/TiO_2_ Core/Shell Nanofibers

After the ALD deposition of 100 nm thick TiO_2_ layers onto the polymer nanofibers, the composites had the same fibrous structure (Figure 1b and Figure 2b). The polymers did not soften during the exothermic ALD reactions. The overall diameter of the PVA/TiO_2_ core/shell nanocomposite was about 300–400 nm, while the PVP/TiO_2_ nanofibers were 600–800 nm.

#### 2.2.2. Thermal Analysis of the Polymer/TiO_2_ Core/Shell Nanofibers in Air

The core was removed from the core/shell composites by two different methods. For the removal by annealing, first the thermal decomposition of PVA and PVP in air had to be investigated by TG/DTA (Figure 3c,d) The steps were similar in air to the ones in nitrogen atmosphere. Again, just water evaporated up to 100 °C and then above 230 °C the polymers started to burn, the decomposition of more overlapping steps [30], than in nitrogen in the case of both polymers. When the polymers were heated in nitrogen, by 600 °C, they did not decompose totally there was some organic char residue that remained; however, in air by 550 °C both the PVA and the PVP burnt out completely. Based on this, for the removal of the core the following heating program was chosen: the samples were heated by 10 °C/min up to 230 °C and then the heating rate was lowered to 2 °C/min until the temperature reached 550 °C. The lowering of the heating rate was necessary in order to protect the as-formed nanotube structure, otherwise the evolved gases would break up the walls.

#### 2.2.3. SEM-EDX of The TiO_2_ Nanotubes

On the SEM images (Figure 1c,d and Figure 2c,d) it was visible that after annealing the PVA/TiO_2_ and the PVP/TiO_2_ nanocomposites, the core was successfully removed and TiO_2_ nanotubes were formed. These had a wall thickness of about 100 nm. The other way for removing the polymer core was dissolution in water. The SEM images (Figure 1e,f and Figure 2e,f) showed similar results to the annealed samples, again tubular structures could be seen. The electrospun nanofibers were several centimeters long and the length stayed the same after the ALD depositions as well; however, after either dissolution or annealing, the nanotubes broke into shorter, few micrometer long pieces.

In all the cases the SEM studies were accompanied by EDX measurements (Table 1). After the deposition the EDX spectra showed the presence of a high amount of Ti in the samples. Also, some Cl content was observable, which implicated that a small amount by-product from the TiCl_4_ precursor remained in the sample. The amount of carbon significantly lowered after both the dissolution and the annealing. Some of the remaining carbon might have come from the double-sided carbon tape, which was used to fix the samples to the sample holders during the measurements. The carbon content was higher after dissolution, than after annealing, which implied that annealing was the more efficient way to remove the polymer core. Also, the TiO_2_ nanotubes prepared from the PVP/TiO_2_ nanofibers had a lower carbon amount, which can be explained by the wider diameter of these fibers, which made the elimination of the polymer easier from the PVP/TiO_2_ composite, than from the one containing PVA.

#### 2.2.4. FT-IR Spectroscopy of The TiO_2_ Nanotubes

**Figure 4:**
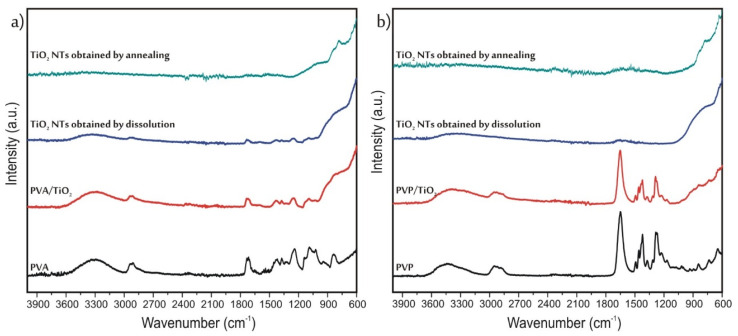
ATR-IR results of the samples prepared from (**a**) PVA, (**b**) PVP.

The SEM-EDX results in themselves were not enough to prove that the polymer was completely removed; therefore, the infrared spectra of the samples were also studied (Figure 4). First, the spectra of the pure PVA (Figure 4a) and PVP (Figure 4b) were measured. In the spectrum of PVA the broad peak between 3100–3500 cm^−1^ referred to the stretching vibrations OH groups, the peaks at 1732 cm^−1^ and 1088 cm^−1^ corresponded to the C=O, while the absorption band at 839 cm^−1^ to C-C bonds. The peaks at 1373 cm^−1^ and 1240 cm^−1^ were respectively the wagging vibrations of the CH2 and CH groups [31]. In the case of PVP again, the band 3100–3500 cm^−1^ signified the stretching vibrations OH groups. At 1460 cm^−1^ and 1422 cm^−1^ the bending modes of the CH2 groups appeared. The absorption bands at 1649 cm^−1^, 1285 cm^−1^ and 1271 cm^−1^ corresponded respectively to stretching vibrations of the C=O, C-O and the C-N bonds [32,33]. After the deposition of TiO_2_ onto the polymers, under 900 cm^−1^ the bands referring to TiO_2_ appeared on the spectra, but the peaks of the polymers were still visible as well. In the case of the composites containing PVP, both after dissolution and annealing, the polymer bands completely disappeared from the spectra. Meanwhile, in the case of PVA, after annealing there was no sign of any remaining polymer on the spectra; however, after dissolution there were still some small peaks of the PVA. These results were in correlation with the EDX measurements, the TiO_2_ nanotubes obtained by dissolution from the PVA/TiO_2_ nanocomposite had the highest carbon content.

#### 2.2.5. Powder XRD Measurement of the TiO_2_ Nanotubes

**Figure 5:**
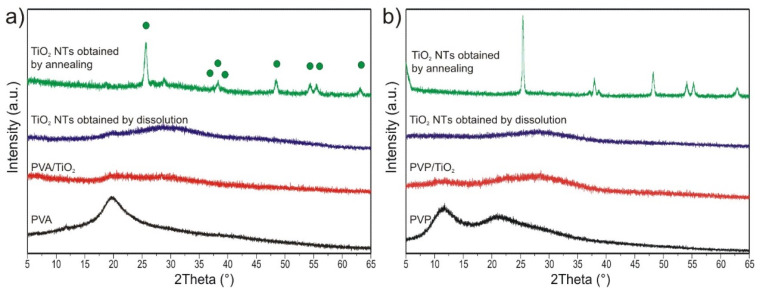
XRD patterns of the samples prepared from (**a**) PVA, (**b**) PVP (The anatase peaks have been now marked with green circles).

The XRD patterns of the samples were recorded as well (Figure 5). The diffractograms showed that both the PVA (Figure 5a) and the PVP (Figure 5b), as well as the ALD deposited TiO_2_ were amorphous. After dissolution no peaks appeared on the diffractograms; hence, as expected the TiO_2_ stayed amorphous. After annealing to 550 °C the TiO_2_ nanotubes crystallized in anatase form, which was identified based on the ICDD database (ICDD-01-075-2547). There is one small peak at Figure 5a, at ca. 28°, which does not match anatase. It might be due to some trace impurities or minor amount of rutile TiO_2_ (ICDD 01-88-1175). The average crystallite size of the anatase TiO_2_ nanotubes prepared from the PVA/TiO_2_ composite was 35–40 nm, while for the one made from PVP/TiO_2_ it was 20–25 nm.

#### 2.2.6. UV-Vis Spectroscopy of the TiO_2_ Nanotubes

**Figure 6:**
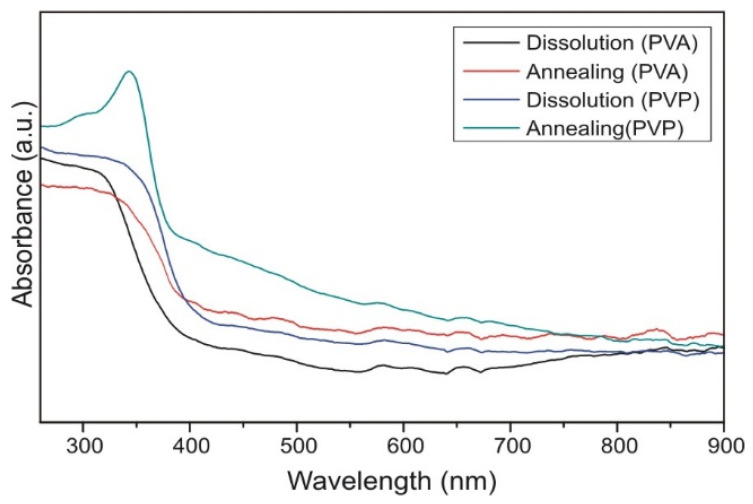
UV-Vis spectra of the TiO_2_ nanotubes.

From the UV-Vis spectra of the TiO_2_ nanotubes (Figure 6) the absorption edges were determined, and from those the band gap energies were calculated [34]. The band gap energy for the samples prepared by dissolution from PVA and PVP were 3.31 eV and 3.15 eV, while for the anatase TiO_2_ nanotubes synthetized by annealing from PVA and PVP were 3.03 eV and 3.10 eV. It is noted that the bandgap of ALD prepared amorphous TiO_2_ nanotubes is wider than the corresponding crystalline ones. This might be unexpected at first sight, because crystalline TiO_2_ is usually tend to have lower concentration of defects, and thus wider bandgap, compared to amorphous TiO_2_. However, recently it was revealed that the bandgap of amorphous TiO_2_ strongly depended on the Ti:O ratio and on the type of defects in the structure. Certain compositions (preferably partially reduced TiO_2_ with oxygen vacancies) resulted in higher bandgap for amorphous TiO_2_ compared to crystalline TiO_2_, as revealed both experimentally and by ab initio calculations [35,36,37].

#### 2.2.7. Photocatalysis Study of the TiO_2_ Nanotubes

**Figure 7:**
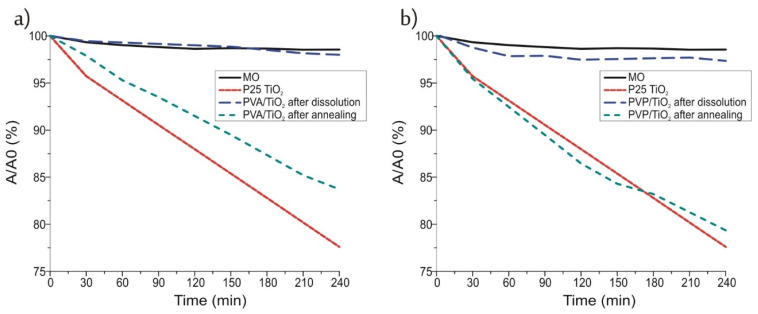
Photocatalysis of the TiO_2_ nanotubes prepared from (**a**) PVA/TiO_2_, (**b**) PVP/TiO_2_ composite.

Finally, the photocatalytic activity of the samples was investigated by decomposing methyl orange dye under UV light and compared to P25 TiO_2_, which was used as a reference (Figure 7). The P25 TiO_2_ decomposed more than 20% of the dye under these circumstances in 4 h.

**Figure 8:**
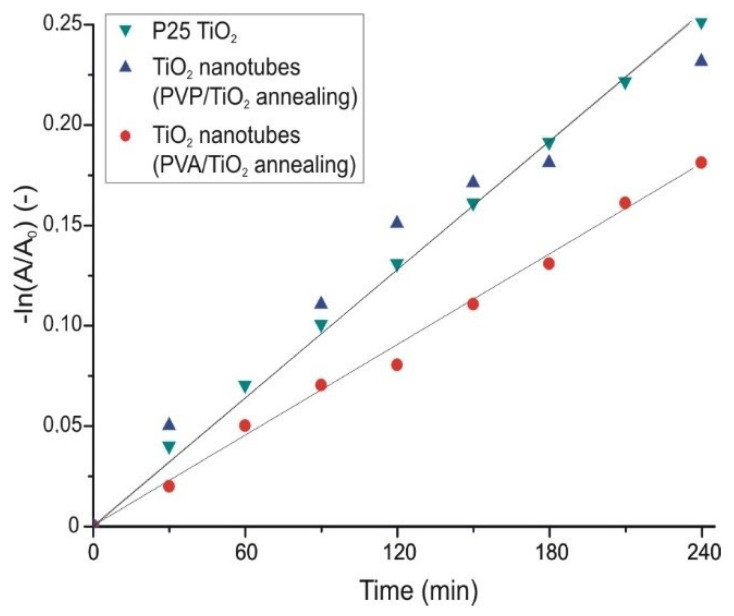
Kinetics of the photocatalytic reaction of the crystalline TiO_2_ nanotubes.

The TiO_2_ nanotubes that were prepared by annealing from the PVA/TiO_2_ composite, had almost as good an efficiency, while the nanotubes prepared by annealing from the composite containing PVP as the core, had the same effect as the P25 TiO_2_. These samples consisted almost completely of anatase TiO_2_. In the case of the crystalline TiO_2_ nanotubes the apparent reaction rate constants (k_app_) were determined as well assuming pseudo first order kinetics (Figure 8) [38]. The constants for the TiO_2_ prepared from the PVA composite was 7.3 × 10^−4^ min^−1^, while for the one prepared from the PVP/TiO_2_ composite and for the P25 TiO_2_ it was 10.5 × 10^−4^ min^−1^.

The photocatalytic activity of the nanotubes prepared by dissolution, clearly did not reach the activity of the annealed samples, mainly because these contained amorphous TiO_2_. However, there was a small but detectable effect in the case of the amorphous TiO_2_ nanotubes prepared from the PVP/TiO_2_ nanofibers. This was probably not observable in the case of the sample prepared by dissolution from the PVA/TiO_2_ composite because that contained a higher amount of remaining residue from the PVA core, and its photocatalytic effect could have been split between decomposing the dye and the remaining polymer [39]. Still in the case of the amorphous TiO_2_ nanotubes prepared from the composite containing PVP, the observed photocatalytic activity was an unexpected.

The photocatalytic feature of ALD prepared amorphous TiO_2_ was first detected when amorphous TiO_2_ was grown onto lotus leaf by ALD at relatively low temperature [7]. Since then the our group has demonstrated the photocatalytic activity of ALD prepared amorphous TiO_2_ by several examples using various substrates such as fullerene, carbon aerogel, SiO_2_ or PMMA nanoparticles, graphene oxide [8,40,41]. For this odd photocatalytic behavior of ALD grown amorphous TiO_2_, several possible reasons were assumed, e.g., (1) the coating interacted with the substrate during the photocatalytic reactions, (2) the samples contained nanocrystalline domains or (3) there was a small amount of carbon atoms left over from the ALD precursor (TTIP) in the TiO_2_ layers. When the photocatalytically inactive SiO_2_ or PMMA were the substrates, amorphous TiO_2_ still had photocatalytic effect. Even in these cases, charge separation between the outer layer and the substrate might contribute to the photocatalytic effect; however, here in the case of TiO_2_ nanotubes the core was completely removed (1). Electron diffraction studies did not reveal nanocrystalline domains, which were previously not visible by XRD (2). In the present study the Ti precursor was carbon free (unlike e.g., TTIP or TDMAT), therefore there were no carbon impurities in amorphous TiO_2_. Although Cl might be present, but it is unlikely to account for the same photocatalytic activity as a supposed C content (3).

Very recent studies on [42,43] ALD grown amorphous TiO_2_ and AL_2_O_3_ layers might provide an explanation for our data about the photocatalytic property of low temperature ALD prepared amorphous TiO_2_. In these studies it was shown that partially reduced, oxygen deficient, amorphous, ALD grown TiO_2_ and Al_2_O_3_ layers had oxygen vacancies and increased amount of non-lattice oxygen in their structure. These could successfully trap holes, and this way they decreased the charge recombination and increased the lifetime of photogenerated charge carriers, and contributed to a higher photocatalytic efficiency.

## 3. Materials and Methods

### 3.1. Preparation of the Polymer Fibers

The poly(vinyl alcohol) (PVA) and the poly(vinylpyrrolidone) (PVP) fibers were prepared by electrospinning, both from their aqueous solutions. For the electrospinning of the PVA fibers 0.4 g of the solid poly(vinyl alcohol) was dissolved in 4 cm^3^ of distilled water. The PVP solution was prepared by dissolving 1.1 g of poly(vinylpyrrolidone) in 4 cm^3^ of distilled water. The PVA solution was stirred for 4 h, while the PVP solution for 24 h, both at room temperature. For the electrospinning, the solutions were transferred into a plastic syringe, which was connected to the needle tip by polymer tubing. The applied voltage was 20 kV and a feeding rate of 0.5 cm^3^/h was used. The nanofibers were collected on aluminum foil, which was covered with a polypropylene fabric. The fabric was applied, because it was easier to remove the fiber mats from them without damaging the polymer nanofibers.

### 3.2. Thermal Analysis of the Polymer Fibers

Before the atomic layer deposition of TiO_2_ could be done, it had to be determined what deposition temperature could be used, so that the polymers would not soften. For this the thermal properties of both the PVA and PVP were investigated in inert atmosphere, which simulates the circumstances of the ALD reactor better (nitrogen, flow rate: 130 cm^3^/min). The thermal analysis was also carried out in oxidative atmosphere (air, flow rate: 130 cm^3^/min) to determine what annealing temperature is needed to totally remove the polymer core. Both measurements were done in a TA Instruments SDT 2960 simultaneous TG/DTA equipment, the polymers were heated with 10 °C/min heating rate to 600 °C in Pt crucibles.

### 3.3. Atomic Layer Deposition of the TiO_2_ Layer

The TiO_2_ thin films were prepared by atomic layer deposition from titanium tetrachloride (TiCl_4_) and H_2_O precursors in a Beneq TFS-200-186 reactor. The pulse time for both precursors was 0.2 s, which was followed by a 3 s nitrogen purge. The layers were grown in 1400 cycles at 50 °C. The theoretical thickness (100 nm) was confirmed by profilometer (AMBIOS XP-I) after the deposition.

### 3.4. Preparation of the TiO_2_ Nanotubes

The removal of the PVA and PVP polymer cores was done by two different methods. Both the PVA and the PVP are water-soluble; thus, one approach was dissolution. For this the samples were put into 60 °C water for 2 h. The sample could not have been stirred, because it would have damaged the nanotubes; hence, the adequate efficiency of the dissolution was achieved by changing the solvent every 30 min. The other method for removing the polymer was annealing, which was done in a TA Instruments SDT 2960 simultaneous TG/DTA instrument. The composites were annealed to 550 °C. At around 230 °C the more intense decomposition of the polymers started; thus, until that temperature the heating rate was 10 °C/min, but after reaching it, the heating rate was lowered to 2 °C/min to avoid the cracking of the oxide walls.

### 3.5. Characterization

The morphology of all the samples was studied by scanning electron microscopy (SEM) with a JEOL JSM-5500LV microscope. The samples were coated by a thin Au/Pd layer in a sputter coater before the measurements were carried out. The composition of the samples was also studied by energy dispersive X-ray (EDX) analysis, the measurements were done at 20 kV. High resolution images were recorded by a LEO 1540 XB scanning electron microscope.

The powder XRD patterns were recorded with a PANalytical X’pert Pro MPD X-ray diffractometer using Cu K_α_ irradiation. The crystallite sizes were calculated based on the Scherrer-equation.

The FT-IR spectra were studied by a Bruker Tensor 37 IR spectrometer equipped with a Goldengate SpecAC ATR head.

The UV-Vis spectra were recorded on an Avantes AvaSpec-2048 spectrophotometer in reflectance mode, using Teflon background.

Finally, the photocatalytic activity of the samples was investigated by decomposing 0.04 mM aqueous solution of methyl orange (MO) dye under UV irradiation. For the measurements 1 mg of the samples was put into 3 cm^3^ of the MO solution in quartz cuvettes. Before the UV illumination, the samples were kept in the dark for 2 h to reach the adsorption equilibrium. After that they were illuminated for 4 h by two parallel UV lamps (Osram blacklight, 18 W, UV-A) that were placed 5–5 cm from the sample. The measured wavelength of the lamp is between 350–390 nm (maximum intensity at 375 nm), and the estimated power at the samples is 0.5 W. The UV-Vis spectra of the MO were measured every 30 min by a Jasco V-550 UV-Vis spectrophotometer. The relative absorbance values were determined at the 464 nm absorption peak of the MO and plotted versus time. As a reference the samples were compared to P25 TiO_2_ (Aeroxide) measured under the same conditions.

## 4. Conclusions

The photocatalytic properties of TiO_2_ nanotubes were investigated in this work. For this, at first, PVA and PVP nanofibers were prepared by electrospinning; and then these were coated with TiO_2_ layers by atomic layer deposition (ALD). The polymer core was removed from the PVA/TiO_2_ and PVP/TiO_2_ nanocomposites by two different methods, dissolution and annealing. This gave the opportunity to compare amorphous and crystalline TiO_2_ nanotubes with the same dimensions and morphology. After the samples were annealed, the TiO_2_ crystallized in anatase phase, and the nanotubes prepared from both PVA and PVP had a good photocatalytic efficiency, reaching that of P25. Regarding the dissolution, as expected, the TiO_2_ shell stayed amorphous. However, in the case of the nanotubes prepared from the composite containing PVP, the samples showed a small but detectable photocatalytic effect.

Hence, the photocatalytic property of amorphous TiO_2_ grown by ALD is now confirmed by using substrate free amorphous TiO_2_ layers, i.e., by the as-prepared amorphous TiO_2_ nanotubes of the present study, complementing our previous data about amorphous ALD TiO_2_ deposited onto various substrates. It seems that the possible partially reduced structure of ALD grown oxides (especially oxygen vacancies and non-lattice oxygens) might be the key, and with them the photocatalytic property of amorphous TiO_2_ can be successfully explained.

It is suggested that these observations can open a very new field for various ALD prepared amorphous oxides (e.g., ZnO, ZrO_2_, SnO_2_, WO_3_, MoO_3_, etc.) as effective photocatalysts. The optimal layer thickness, composition, etc. have to be investigated further for each oxide, and more insight is needed into the occurring phenomena as well. It is also supposed that other synthesis methods (e.g., sol-gel, hydrothermal, sputtering, etc.) can also yield similarly active photocatalytic amorphous oxides. These can lead to novel applications and products, e.g., coating highly structured heat sensitive substrates with cheap, biocompatible, self-cleaning, ultrathin films.

## Figures and Tables

**Table 1:** EDX results of the composites.

		Samples Containing PVA			Samples Containing PVA		
		PVA/TiO_2_	TiO_2_ NTs Dissolution	TiO_2_ NTs Annealing	PVP/TiO_2_	TiO_2_ NTs Dissolution	TiO_2_ NTs Annealing
C	%	36.4	15.7	6.0	39.0	7.5	4.6
N		-	-	-	7.5	3.3	2.1
O		38.3	45.6	59.1	28.8	49.2	46.5
Cl		2.6	4.3	1.0	3.3	5.1	1.3
Ti		22.7	34.4	33.9	21.4	34.9	45.5

## Sample Availability

Samples of the compounds are not available from the authors.
